# GLUT-1 Enhances Glycolysis, Oxidative Stress, and Fibroblast Proliferation in Keloid

**DOI:** 10.3390/life11060505

**Published:** 2021-05-30

**Authors:** Ying-Yi Lu, Chieh-Hsin Wu, Chien-Hui Hong, Kee-Lung Chang, Chih-Hung Lee

**Affiliations:** 1 Department of Dermatology, Kaohsiung Veterans General Hospital, Kaohsiung 81362, Taiwan; u103800011@cc.kmu.edu.tw (Y.-Y.L.); chhong@vghks.gov.tw (C.-H.H.); 2 Graduate Institute of Medicine, College of Medicine, Kaohsiung Medical University, Kaohsiung 80708, Taiwan; 3 Department of Health and Beauty, Shu-Zen Junior College of Medicine and Management, Kaohsiung 821, Taiwan; 4 Division of Neurosurgery, Department of Surgery, Kaohsiung Medical University Hospital, Kaohsiung 807, Taiwan; 940065@ms.kmuh.org.tw; 5 Department of Surgery, School of Medicine, College of Medicine, Kaohsiung Medical University, Kaohsiung 80708, Taiwan; 6 Department of Dermatology, School of Medicine, National Yang Ming Chiao Tung University, Taipei 112, Taiwan; 7 Department of Biochemistry, School of Medicine, College of Medicine, Kaohsiung Medical University, Kaohsiung 80708, Taiwan; 8 Department of Medical Research, Kaohsiung Medical University Hospital, Kaohsiung 807, Taiwan; 9 Department of Dermatology, Kaohsiung Chang Gung Memorial Hospital and Chang Gung University College of Medicine, Kaohsiung 83301, Taiwan

**Keywords:** glucose transporter 1 (GLUT-1), glycolysis, keloids, proliferation, reactive oxygen species (ROS)

## Abstract

A keloid is a fibroproliferative skin tumor. Proliferating keloid fibroblasts (KFs) demand active metabolic utilization. The contributing roles of glycolysis and glucose metabolism in keloid fibroproliferation remain unclear. This study aims to determine the regulation of glycolysis and glucose metabolism by glucose transporter-1 (GLUT-1), an essential protein to initiate cellular glucose uptake, in keloids and in KFs. Tissues of keloids and healthy skin were explanted for KFs and normal fibroblasts (NFs), respectively. GLUT-1 expression was measured by immunofluorescence, RT-PCR, and immunoblotting. The oxygen consumption rate (OCR) and extracellular acidification rate (ECAR) were measured with or without WZB117, a GLUT-1 inhibitor. Reactive oxygen species (ROS) were assayed by MitoSOX immunostaining. The result showed that glycolysis (ECAR) was enhanced in KFs, whereas OCR was not. GLUT-1 expression was selectively increased in KFs. Consistently, GLUT-1 expression was increased in keloid tissue. Treatment with WZB117 abolished the enhanced ECAR, including glycolysis and glycolytic capacity, in KFs. ROS levels were increased in KFs compared to those in NFs. GLUT-1 inhibition suppressed not only the ROS levels but also the cell proliferation in KFs. In summary, the GLUT-1-dependent glycolysis and ROS production mediated fibroblast proliferation in keloids. GLUT1 might be a potential target for metabolic reprogramming to treat keloids.

## 1. Introduction

A keloid is a proliferative scar that extends beyond the original injury borders with excess fibroblast proliferation and increased collagen production [1]. It appears as a red, indurated, disfiguring tumor and may be accompanied by intractable pruritus, pain, and even contractions. They tend to develop in high tension skin areas after minor traumas in individuals with a dark skin color [2,3,4]. Currently available treatments for keloids are palliative in nature [5] and they tends to recur despite therapeutic interventions [6,7], causing profound functional and psychosocial sequelae [8]. This therapeutic challenge is partly attributed to the poor understanding of its pathogenesis. Several etiological factors are proposed to contribute to keloid development, including abnormal fibroblast proliferation, overactivated myofibroblasts, aberrant immune regulations, dysregulated mechanical forces, and epithelial-to-mesenchymal transition (EMT) [4,9,10]. During the development of keloids, excessive fibroblast proliferation, myofibroblast activation, and collagen production all demand sufficient cellular energy for cells to proceed [11,12,13]. Increased inflammation and mechanical properties also regulate energy production to match the increased energy consumption [14,15,16]. For example, positron emission tomography (PET) images in patients with keloids demonstrate that fluorodeoxyglucose (FDG) increases to compensate for increases in glucose uptake and energy consumption [17,18].

Glucose metabolism is essential for a continuous supply of energy to active cells. Glycolysis is the crucial pathway in glucose metabolism, in which glucose uptake serves as a first and critical niche for facilitating the basal glucose uptake across the cell membrane, and for ensuring the viable flux of glucose for further metabolisms [19,20,21,22,23]. In mammalian cells, glucose transporter-1 (GLUT-1) is the most highly conserved and widely distributed glucose transporter [24]. The GLUT-1 functions as a rate-limiting step in the flux of glucose for glycolysis and cell proliferation. The over-expression of GLUT-1 was associated with growth and poor prognosis in several human tumors [25], including breast cancer and prostate cancer [26,27]. For example, in breast cancer, GLUT-1 controls the rate of glycolysis, glucose uptake, and lactate secretion [28]. In prostate cancer, GLUT-1 regulates glycolysis and cell proliferation [29,30].

In addition to glycolysis, cellular energy homeostasis requires a good coordination of mitochondrial oxidative phosphorylation (OXPHOS) and glycolysis to be maintained [31,32]. In normal cells, metabolic activities mainly depend on OXPHOS rather than glycolysis to generate adenosine triphosphate (ATP) for energy production. OXPHOS and glycolysis constitute a tightly coupled and coordinated interconversion system [33,34]. The attributed proportion of OXPHOS to glycolysis for energy production varies in different cells, growth states, and microenvironments [33]. In highly proliferative cells or in hypoxia conditions, glycolysis is enhanced and OXPHOS capacity is reduced [22]. Inhibition of OXPHOS triggers a rapid compensatory increase in glucose flux through the upregulation of GLUT-1 [35]. Authors reported that keloid fibroblasts (KFs) act like cancer cells, a Warburg effect, for energy utilization by a great reliance on glycolysis, not on OXPHOS for ATP production [36,37]. KFs tend to undergo a metabolic reprogramming with enhanced glucose uptake and lactate production as compared to normal fibroblasts (NFs) [36,38,39].

During energy production, ROS is generated inside the cells. Inside the cell, mitochondrial OXPHOS is the predominant source of reactive oxygen species (ROS), mainly from the electron transport chain (ETC) [40]. Several studies reported that ROS are increased in keloids, suggesting that the increased oxidative stress may contribute to keloid formation [41,42]. An elevated GLUT-1 level in keloids has also been reported [43]. However, how GLUT-1 regulates ROS production in keloids remains unknown. Moreover, the functional and mechanistic role of GLUT-1 in keloid pathogenesis, including glycolysis, OXPHOS, and oxidative stress have not been clarified. In this study, we aim to determine the regulation of the glycolysis and OXPHOS in keloids and the mechanisms by which GLUT-1, an essential protein to initiate cellular glucose uptake, controls the coordination of glycolysis, OXPHOS, and oxidative stress.

## 2. Materials and Methods

### 2.1. Subjects Involved

All participating individuals were Taiwanese of Han Chinese origin over the age 20 years and visited the Department of Dermatology at Kaohsiung Veterans General Hospital between 2016 and 2019. In this study, participants with keloids (n = 6) and healthy controls (n = 6) were recruited (Table 1). A keloid was defined as a skin lesion exhibiting continuous growth beyond the margin 6 months after trauma or surgery. Patients were excluded if they received treatment with intralesional corticosteroid injection or liquid nitrogen therapy within the past 3 months or they had associated invasive cancers. Tissue samples were taken from the center of the keloid with a 3-mm biopsy punch. Control samples of normal skin were obtained from perilesional skin after elective surgical excision for melanocytic nevus or epidermal cysts. The protocol of this study was approved by the Institutional Review Board of Kaohsiung Veterans General Hospital (VGHKS16-CT5-10).

### 2.2. Cell Culture and Treatment

Human fibroblasts were isolated from dermal tissues of control or keloid skin. Dermal tissues were cut to 1–2 mm^3^; then, fibroblasts were cultured in DMEM (12100046, Gibco, Waltham, MA, USA) medium supplemented with 10% fetal bovine serum (FBS) (A4766801, Gibco, Waltham, MA, USA) and 1% penicillin-streptomycin (P/S) (15140122, Gibco, Waltham, MA, USA), and maintained at 37 °C humidified air with 5% CO_2_. Fibroblasts with the third to sixth passage were used for the experiments. Three strains of KFs and three strains of NFs derived from punched tissue samples were used in the study. To examine the role of GLUT-1 on NFs and KFs, fibroblasts were starved in serum-free DMEM for at least 12 h before treatment using 10 µM WZB117, a GLUT-1 inhibitor (6143, PeproTech, Rocky Hill, NJ, USA) for 48 h.

### 2.3. Seahorse Analysis for OCR and ECAR

**Figure 1:**
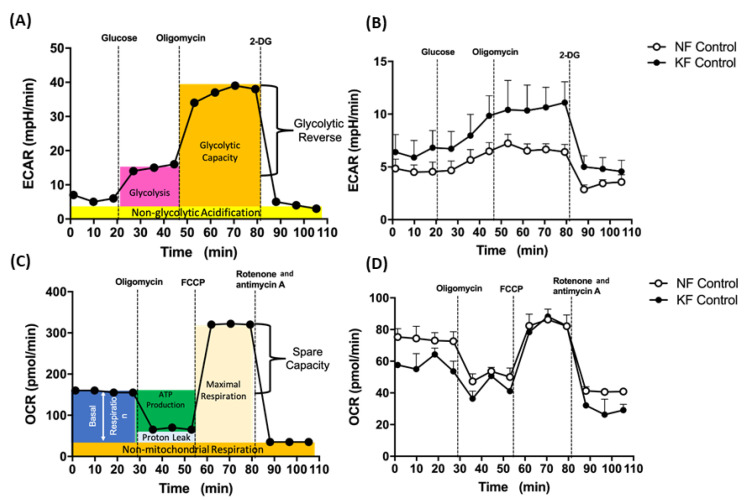
Enhanced glycolysis in keloid fibroblasts (KF). (**A**) The continuous ECARs and OCRs were recorded and analyzed using an XF94 extracellular flux analyzer. The scheme of ECARs shows the section of the time trace corresponding to each module. Subsequently, 10 mM glucose, 1 µM oligomycin, and 75 mM 2-DG were injected to the medium. (**B**) Representative ECAR was measured in KFs and NFs (1 × 10^4^ cells/well). Data are presented as mean ± SEM. (N = 6, * *p* < 0.05, ** *p* < 0.01, *** *p* < 0.001.). The representative data from 3 independent experiments are shown. (**C**) Continuous OCRs values were recorded and analyzed. The scheme of continuous of OCRs shows the section of the time trace corresponding to each module. Subsequently, 1 µM oligomycin, 1 µM FCCP, and 0.5 µM rotenone/antimycin A were injected to the medium. (**D**) Representative OCRs were measured in KFs and NFs (1 × 10^4^ cells/well). Data are presented as mean ± SEM (N = 6, * *p* < 0.05, ** *p* < 0.01, *** *p* < 0.001.). Shown is the representative data from 3 independent experiments.

A Seahorse XF24 Extracellular Flux Analyzer (Agilent, Santa Clara, CA, USA) was used to continuously monitor the extracellular acidification rate (ECAR) and oxygen consumption rate (OCR) of fibroblasts. An XF Glycolysis Stress Test kit (103020, Agilent, Santa Clara, CA, USA) was used for glycolysis and an XF Mito Stress Test kit (103015-100, Agilent, Santa Clara, CA, USA) was used to test mitochondrial stress. Fibroblasts (1 × 10^4^ cells /well) were seeded in an XF24 culture plate in DMEM supplemented with 2% FBS at 5% CO_2_ at 37 °C for 18–20 h. Before the experiment, fibroblasts were washed with PBS, replaced with 625 µL DMEM (pH = 7.4) without FBS and sodium bicarbonate, and then incubated in a CO_2_-free incubator at 37 °C. For the ECAR assay, 10 mM glucose, 1 µM oligomycin, and 75 mM 2-deoxy-glucose (2-DG) (a glucose analog) in XF Glycolysis Stress Test kit (103020, Agilent, Santa Clara, CA, USA) were injected to the medium (Figure 1A). First, glucose was injected to induce glycolysis. Subsequent treatment with oligomycin shut down OXPHOS with a compensatory glycolytic capacity. Next, by inhibiting glycolysis with 2-DG, glycolysis reserve was estimated by the glycolysis capacity divided by basal ECAR. For the OCR assay, 1 µM oligomycin (a complex V inhibitor), 1 µM carbonyl cyanide-phospho-(p)-trifluoro-methoxyphenyl-hydrazon (FCCP) (a proton gradient uncoupler), and 0.5 µM rotenone (a complex I inhibitor)/antimycin A (a complex III inhibitor) in an XF Mito Stress Test kit (103015-100, Agilent, Santa Clara, CA, USA) were subsequently injected to the medium (Figure 1C). First, baseline cellular OCR was measured, from which basal respiration was derived by subtracting non-mitochondrial respiration. Next, oligomycin was added, and the resulting OCR was used to derive ATP-production respiration (by subtracting the oligomycin rate from baseline cellular OCR) and proton-leak respiration (by subtracting non-mitochondrial respiration from the oligomycin rate). Further, FCCP was added to collapse the inner membrane gradient, driving the ETC to function to its maximal rate, and maximal respiratory capacity was derived by subtracting non-mitochondrial respiration from the FCCP OCR. Lastly, ETC function was shut down by addition of rotenone and antimycin A, which revealed the non-mitochondrial respiration. The mitochondrial spare capacity was calculated by subtracting basal respiration from maximal respiratory capacity.

### 2.4. Immunohistochemistry (IHC) for GLUT-1 In Tissue

Paraffin-embedded samples of keloid and normal tissue were cut and mounted on coated slides. After de-paraffinization in xylene and rehydration with graded alcohol solutions, antigen retrieval was performed in sodium citrate buffer (100 °C, 20 min). The Novolink Polymer Detection System (RE7150-K, Leica, Wetzlar, Germany) was carried out according to the manufacturer’s introduction. The sections were neutralized with endogenous peroxidase using a Peroxidase Block for 5 min, blocked by incubation with Protein Block (RE7102, Leica, Wetzlar, Germany) for 5 min, then incubated overnight at 4 °C with mouse monoclonal antibody anti-GLUT-1 (1:100 dilution, sc3772282, Santa Cruz, CA, USA). After rinsing by phosphate buffered saline (PBS) (11-223-1M, Biological Industries, Cromwell, CT, USA), the slides were incubated with Novolink Polymer for 30 min. Afterwards, the sections were developed with 3,3′-diaminobenzidine (DAB) working solution, counterstained with hematoxylin for 5 min, dehydrated, and mounted by mounting medium. Finally, 6 view fields were randomly selected on each section and observed under a microscope (Olympus, Tokyo, Japan) to determine the expression.

### 2.5. Immunofluorescence Assay (IFA) for GLUT-1 and Ki-67 in KFs and NFs

For cell staining, fibroblasts were plated at 1 × 10^5^ cells per well in 12-well plates and incubated overnight. After treatment with WZB-117, fibroblasts were fixed with 4% paraformaldehyde for 15 min, then permeabilized in PBS with 0.1% Triton X-100 for 5 min. The fixed cells were blocked with 10% skim milk in PBS, incubated overnight with anti-GLUT-1 antibody (1:100 dilution, 12939, Cell Signaling, Danvers, MA, USA) or anti-Ki67 antibody (1:1000 dilution, ab16667, Abcam, Cambridge, England) at 4 °C, washed repeatedly with PBS, and finally replaced in secondary antibodies conjugated with Alexa Flour 488 dye (1:1000 dilution, A11001, Invitrogen, Waltham, MA, USA) and incubated for 2 h at room temperature. After DAPI staining (1:5000 dilution, 62248, Thermo Fisher, Waltham, MA, USA) for 15 min, sections of stained cells were visualized and examined under a fluorescence microscope (BX53 system microscope, OLYMPUS, Tokyo, Japan). The mean intensity of GLUT-1 and Ki-67 was determined from 5 randomly selected fields of each tissue section through Image J (National institutes of Health, Bethesda, MA, USA).

### 2.6. RNA Extraction, Reverse Transcriptase PCR (RT-PCR), and Quantitative Real Time PCR (qPCR)

The total RNA from fibroblasts was extracted using 1 mL TRIzol reagent (15596018, Invitrogen, Waltham, MA, USA). After the addition of 200 µL 1-Bromo-3-chloropropane (B9673, Sigma, St. Louis, MO, USA), RNA was precipitated with 500 µL isopropanol (278475, Sigma, St. Louis, MO, USA) and pelleted by centrifugation at 13,000× rpm at 4°C for 20 min. The pelleted RNA was washed with 1 mL 75% ethanol, then centrifuged at 13,000× rpm at 4 °C for 10 min. After removal of the supernatant, it was air-dried for 6 min and suspended in nuclease-free water. The RNA concentration was determined by Spectrophotometer (Eppendorf, Hamburg, Germany). For RT-PCR, 1.5 μg samples of total RNA were reverse transcribed into cDNA in 20 μL of reaction mixture containing 200 units of Superscript III enzyme, 0.5 μL random primer, 0.5 μL 25 mM dNTP, 4 μL 5× first strand buffer, 1 μL 0.1 M dithiothreitol (DTT), and 1.8 μL H_2_O in a buffer. The RT procedure was performed according to the protocol suggested by the Superscript III manufacturer (18080085, Invitrogen, Waltham, MA, USA). The resulting cDNA was frozen at −20 °C for mRNA detection. Target genes were amplified by PCR performed with fast SYBR green PCR Master mix (4385612, Thermo Fisher, Waltham, MA, USA). Next, 3 μL cDNA from RT was replicated in PCR reactions in a total volume of 10 μL containing 5 μL fast SYBR Master mix, and 2 μL of the target gene’s forward and reverse primers mix at 3 μM. The PCR conditions were 1 cycle of 95 °C for 20 s followed by 40 cycles of 95 °C for 3 s and 60 °C for 30 s. After completion of PCR, a final melting curve was performed by denaturation at 95 °C for 15 s and then recorded by cooling to 60 °C with slowly heating to 95 °C for 15 s. The RT-PCR was performed on an ABI StepOnePlus Real-Time PCR System (Life Technologies, Waltham, MA, USA). The cDNA was amplified with primers manufactured by Genomics (New Taipei City, Taiwan) (Table 2). The β-actin was used as the internal reference.

### 2.7. Mitochondrial Superoxide Detection

The MitoSOX^TM^ Red mitochondrial superoxide indicator kit (36008, Thermo Fisher, Waltham, MA, USA) was applied to measure mitochondrial superoxide in fibroblasts. Fibroblasts (1 × 10^5^ cells/well) were seeded overnight in a 12 well-plate then incubated with 1 mL 5 µM MitoSOX reagent solution for 10 min at 37 °C in a 5% CO_2_ incubator protected from light. After staining, the cells were replaced with fresh culture medium and observed under a microscope (Olympus, Tokyo, Japan) to determine the superoxide production from mitochondria.

### 2.8. Cell Proliferation Assay

Proliferation was measured by WST-1 Proliferation Assay (11644807001, Roche, Basel, Switzerland). Fibroblasts were seeded at 3 × 10^3^ cells/well (100 μL) in 96-well plates in DMEM with 2% FBS overnight. Fibroblasts were treated with WZB117 at indicated concentration in <2% FBS DMEM and determined at indicated time points. The cell medium was replaced with the WST-1 reagent and then incubated at 37 °C for 2 h. Absorbance was monitored with an Epoch^TM^ 2 Microplate Spectrophotometer (BioTek, Winooski, VT, USA) at 450 nm with a reference wavelength of 620 nm.

### 2.9. Statistical Analysis

All analyses were conducted with Prism 6.0 (GraphPad Software). All other values were expressed as either means ± standard error in the mean (SEM) in three independently performed experiments or as a representative value obtained in three independently performed experiments. Group differences were evaluated by Student’s *t*-test or Mann–Whitney U test. A *p* < 0.05 was considered statistically significant.

## 3. Results

### 3.1. Enhanced Glycolysis in KF

To investigate the mitochondrial energy metabolism in keloids, we first analyzed ECARs and OCRs, which are surrogates of glycolysis and oxidative mitochondrial activity, respectively. The ECAR was measured by sequential injections of glucose, oligomycin, and 2-DG with an XF94 Flux analyzer (for details, see Figure 1A in Methods). Our data showed that, in comparison with NFs, KFs had higher ECARs including rates of glycolysis, glycolytic capacity, and glycolysis reserve (Figure 1B). Next, OCR was measured with an XF94 Flux analyzer after sequential injections of oligomycin, FCCP, and rotenone/actimycin A (for details, see Figure 1C and in Methods). On the other hand, the dynamic components of OCR, including the basal respiratory OCR, proton-leak OCR, ATP production OCR, and maximal respiration OCR were similar among KFs and NFs (Figure 1D). These results suggest that the KFs showed a tendency towards glycolysis.

### 3.2. Glycolytic Enzymes Were Upregulated in KFs

**Figure 2:**
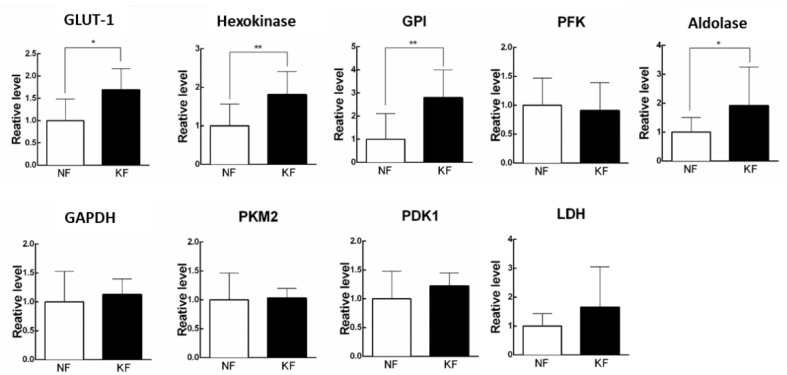
Glycolytic enzymes were upregulated in KFs. RNA was purified and the mRNA level of glycolytic enzymes was determined by RT-PCR. Data are presented as mean ± SEM. (N = 6, * *p* < 0.05, ** *p* < 0.01) from representative data of 3 independent experiments. Abbreviations: GLUT-1, glucose transporter 1; GPI, phosphoglucose isomerase; PFK, phosphofructokinase; GAPDH, glyceraldehyde 3-phosphate dehydrogenase; PKM2, pyruvate kinase M2; PDK1, pyruvate dehydrogenase kinase 1; LDH, lactate dehydrogenase.

Since increased ECARs in the KFs was associated with a reciprocal decrease in OCR, we next asked the mechanism by which ECAR (glycolysis) is enhanced in KFs. To address that, we measured the expression of enzymes in glycolytic pathway, including GLUT-1, hexokinase, GPI, PFK, aldolase, PKM2, LDH, and PDK1 by RT-PCR in KFs (Figure 2A). The results showed that GLUT-1, hexokinase, GPI, and aldolase were significantly upregulated at 1.5-fold, 2-fold, 3-fold, and 2-fold in KFs, respectively, as compared with those in NFs, suggesting that the induction of GLUT-1, hexokinase, GPI, and aldolase may contribute to the high glycolytic phenotype in KFs (Figure 2B).

### 3.3. GLUT-1 Expression Was Increased in Both KFs and Hyperfibrotic Regions in Keloid Tissues

**Figure 3:**
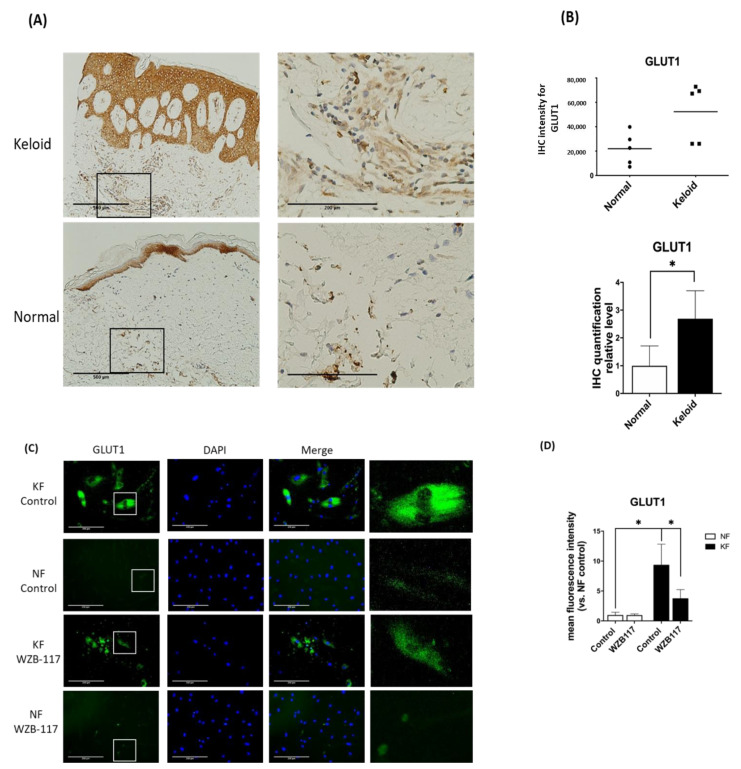
GLUT-1 expression was increased in both Fs and hyperfibrotic regions in keloid tissues. (**A**) Immunohistochemically staining for GLUT-1 in normal skin, and keloids (N = 5, normal skin; N = 5, keloids). Scale bar = 500 μm. Right panels are high-power views of tissues. Scale bar = 200 μm. (**B**) Scatterplots of expression levels; the middle line represents the mean value (* *p* < 0.05). (**C**) The immunofluorescence staining for GLUT-1 in NFs and KFs pretreated with or without 10 µM WZB117 for 48 h. Scale bar = 200 μm. The white rectangle in (**C**) indicates the GLUT-1 morphology for high magnifications at right panels. (**D**) is the quantification of IF from (**C**). Five random fields of each section were selected and more than 35 cells of each field were counted. Data are presented as mean ± SEM from 3 or more independent experiments (N = 6, * *p* < 0.05).

Since GLUT-1 is the key and initial enzyme in the glycolysis and it was found to be significantly increased in KFs, we asked whether GLUT-1 expression is increased in keloid tissues. We then measured the GLUT-1 expression in keloids and normal skin tissues by immunohistochemical analysis. The results showed that the expression of GLUT-1 was present in the epidermis from both keloids and control skin (Figure 3A). Interestingly, in the dermis of the keloids, the expression of GLUT-1 was markedly increased with high collagen deposits as compared with that in the control skin (Figure 3B). In parallel, the expression of GLUT-1 in fibroblasts was examined by immunofluorescence staining. The GLUT-1 expression was distributed along the plasma membrane and throughout the cytoplasm of fibroblasts (Figure 3C). Notably, GLUT-1 expression was nearly 7-fold higher in KFs compared to NFs (Figure 3D). Interestingly, treatment of KFs with WZB117, a GLUT-1 inhibitor, substantially decreased the elevated level of GLUT-1 in KF (Figure 3D). Taken together, these results suggest that GLUT-1 may contribute to the development of fibrotic lesions in keloids.

### 3.4. GLUT-1 Expression Is Critical for Glycolysis but Not Mitochondrial OXPHOS in KFs

**Figure 4:**
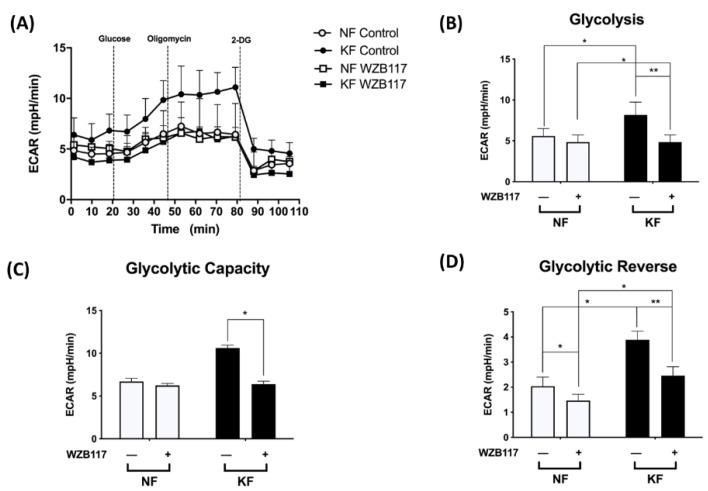
GLUT-1 mediated the enhanced glycolysis in KFs. NFs and KFs were pretreated with or without 10 µM of WZB117 for 48 h and then incubated with base medium at 37 °C in a non-CO_2_ incubator. Subsequently, 10 mM glucose, 1 µM oligomycin, and 75 mM 2-DG were injected to the medium. The ECARs were recorded and analyzed by the Seahorse XF-24 software, including (**A**) changes in the glycolytic function, (**B**) glycolysis, (**C**) glycolytic capacity, and (**D**) glycolytic reserve. Data are presented as mean ± SEM from 3 or more independent experiments. (N = 6, * *p* < 0.05, ** *p* < 0.01).

We noted the increased GLUT-1 expression and increased glycolysis in KFs. We then asked whether GLUT-1 regulates glycolysis in keloids. The ECARs were measured in KFs and NFs with and without treatment with WZB117, a GLUT-1 inhibitor (Figure 4A). Before treatment, the KFs had significantly increased ECARs in terms of glycolytic ATP output (Figure 4B), glycolytic capacity (Figure 4C), and glycolytic reserve (Figure 4D), which is consistent with previous experiments (Figure 1). After treatment with WZB117, the enhanced glycolysis, glycolytic capacity, and glycolysis reserve were significantly suppressed in KFs (Figure 4B–D), indicating that GLUT-1 regulates enhanced glycolysis in keloids.

**Figure 5:**
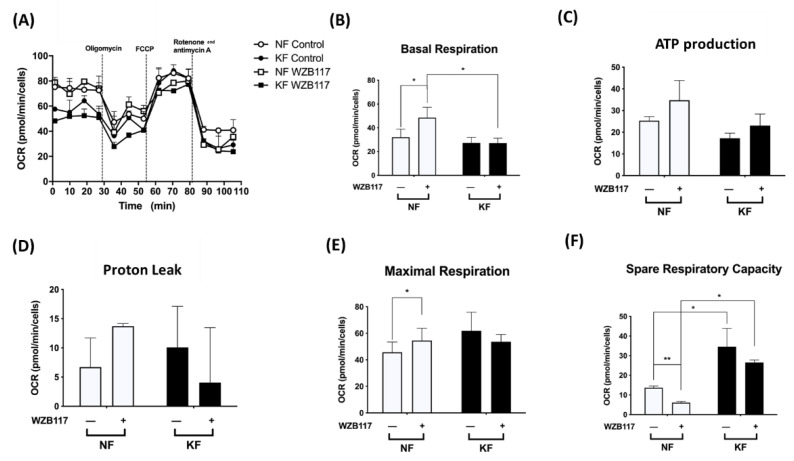
GLUT-1 inhibition increased the mitochondrial OXPHOS in NFs. NFs and KFs were pretreated with or without 10 µM of WZB117 and then incubated at 37 °C in a non-CO_2_ incubator. Subsequently, 1 µM oligomycin, 1 µM FCCP, and 0.5 µM rotenone/antimycin A were injected to the medium. The OCRs were recorded and analyzed by the Seahorse XF-24 software, including (**A**) changes in the mitochondrial respiration, (**B**) basal respiration, (**C**) ATP production, (**D**) proton leak, (**E**) maximal respiration, and (**F**) spare respiratory capacity. Data are presented as mean ± SEM from 3 or more independent experiments. (N = 6, * *p* < 0.05, ** *p* < 0.01, *** *p* < 0.001).

Next, we asked whether GLUT-1 regulated mitochondrial OXPHOS, an indicator of oxidative stress, in KFs. We measured the OCRs in KFs and NFs with or without WZB117 (Figure 5A). Consistent with our previous experiments (Figure 1), KFs and NFs had comparable OCRs, including basal respiration OCR (Figure 5B), ATP-production OCR (Figure 5C), proton-leak OCR (Figure 5D), and the maximal respiration OCR (Figure 5E). However, our further studies showed that spare respiratory capacity of KFs was nearly 3-fold higher than that of NFs (Figure 5F).

After treatment with WZB117 in NFs, the basal respiration OCR (2-fold) and maximal respiration OCR were significantly increased. Although NFs also showed increased ATP production OCR and proton-leak OCR, the increase did not reach statistical significance. Additionally, whereas WZB117 treatment suppressed spare respiration capacity in both NFs and KFs (Figure 5F), the spare respiratory capacity was higher in KFs despite WZB117 treatment. Taken together, these results suggest that KFs are fueled predominantly by GLUT-1 dependent glycolysis rather than mitochondrial OXPHOS. That is, KFs behave toward the Warburg-like effect to meet the energetic demand of fibroproliferation property, in which GLUT-1 has a critical role.

### 3.5. Blocking GLUT-1 Decreased the Enhanced ROS Formation in KFs

**Figure 6:**
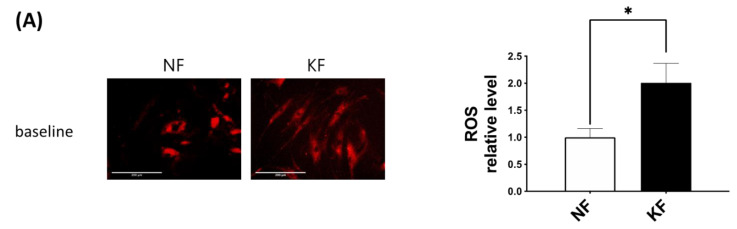

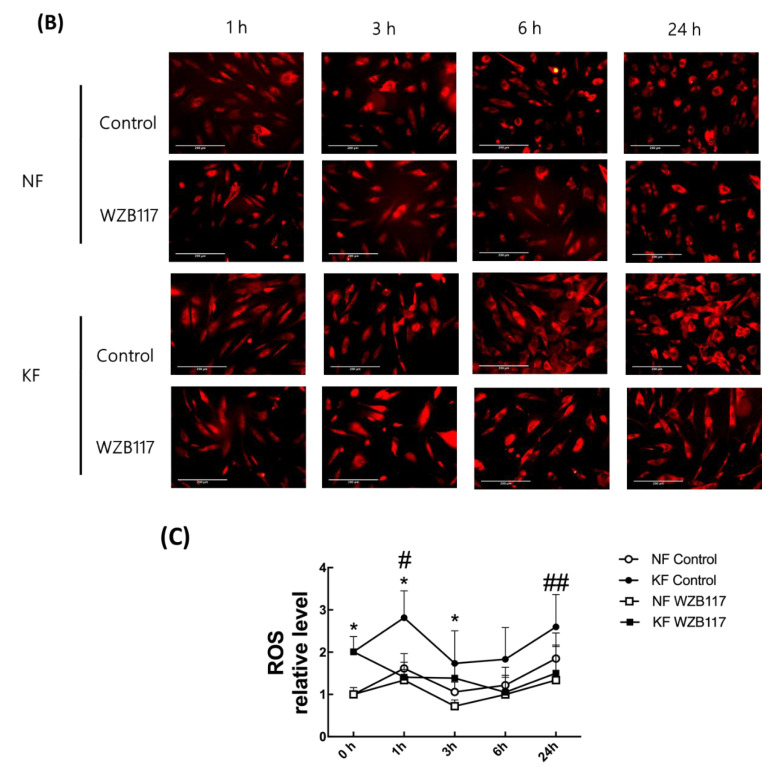
GLUT-1 regulated the enhanced ROS development in KFs. NFs and KFs were pretreated with or without 10 µM of WZB117 for 48 h. (**A**) The ROS at baseline. (**B**) The ROS in NFs and KFs after treatment with WZB117. Measures shown are the representative data from 3 independent experiments. Scale bar = 200 μm. (**C**) is the quantification of relative level from (**B**). Five random fields of each section were selected and more than 50 cells of each field were counted. Data are presented as mean ± SEM (N = 6, * *p* < 0.05, KFs control compared with NFs control; # *p*< 0.05, ## *p* < 0.01, KFs WZB117 compared with KFs control).

The oxidative state is maintained by a balance between antioxidant and pro-oxidant levels. To determine whether GLUT-1 regulated ROS levels, MitoSox was used as an indicator of ROS in NFs and KFs, when they were treated with or without WZB117 (Figure 6A–C). We traced the ROS fluorescence signaling at different time points in each group and counted more than 50 cells in each field to quantify the signals. The results showed that, in the baseline, KFs showed a nearly 2-fold increase of ROS levels compared to those in NFs (Figure 6A). While WZB117 treatment did not alter the ROS levels in NFs after one or 24 h, WZB117 treatment suppressed ROS levels in KFs after 1 or 24 h. In addition, the ROS levels in KFs returned to the baseline ROS levels as in NFs (Figure 6B,C). These data indicated that GLUT-1 mediated enhanced ROS levels, creating oxidative stress in keloids.

### 3.6. GLUT-1 Is Required for Proliferation of KFs

**Figure 7:**
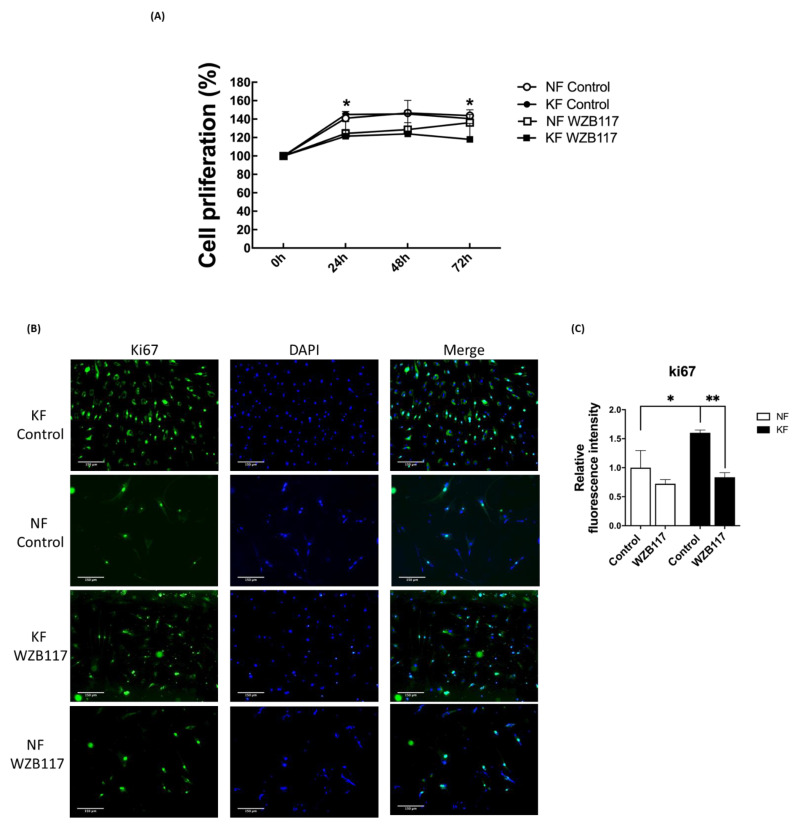
Blocking GLUT-1 decreased proliferation of KFs and NFs, to a greater extent with KFs. NFs and KFs were pretreated with or without 10 µM of WZB117 for 48 h. (**A**) The WST-1 assay was used to determine cell proliferation and cell viability at different time-points. (N = 6, * *p* < 0.05, KFs WZB117 compared with KFs control, NFs WZB117 compared with NFs control). (**B**) The immunofluorescence staining for Ki67+cells in NFs and KFs showed that WZB117-treated fibroblasts had decreased proliferation rates compared controls. Data shown are representative from 3 independent experiments. Scale bar = 150 μm. (**C**) is the quantification of Ki67+ fibroblasts from (**B**). Five random fields of each section were selected and more than 30 cells of each field were counted. Data are presented as mean ± SEM of 3 or more independent experiments (N = 6, * *p* < 0.05, ** *p* < 0.01).

In our previous experiments (Figure 5F), spare respiratory capacity, a measure of the ability of the mitochondria to respond to physiological stress, was enhanced in KFs regardless of GLUT-1 inhibition. We were interested in whether cell proliferation of KFs would be regulated by GLUT-1. To evaluate the effect of GLUT-1 on the cell proliferation in keloids, cell viability and proliferation were measured after WZB117 treatment. At baseline, KF exhibited a slight increase in the cell viability compared to NFs in accordance with the increased spare respiratory capacity. WZB117 treatment suppressed cell viability in both KFs and NFs, although the cell viability in KFs was reduced to a greater extent than that in NF (Figure 7A). To consolidate this finding, further proliferation analysis was performed using Ki-67 labeling (Figure 7B). WZB117-treated KFs showed a significantly reduced Ki-67 intensity compared with controls (Figure 7C), suggesting that GLUT-1 regulates fibroblast proliferation, particularly in keloids.

## 4. Discussion

**Figure 8:**
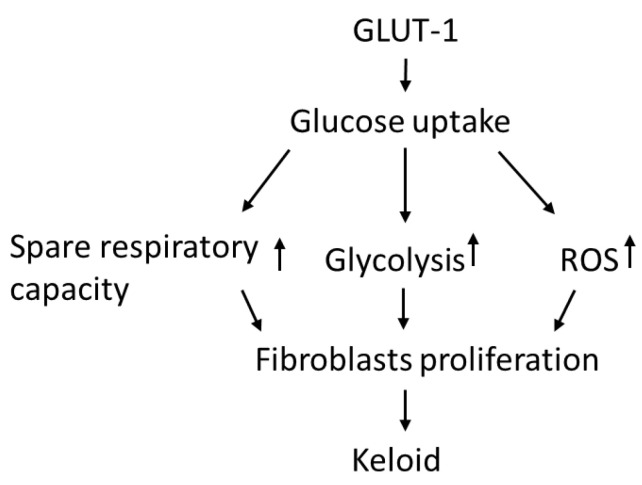
The hypothesized regulatory role of GLUT-1 in glycolysis and metabolic reprograming in the pathogenesis of keloids.

In this study, KFs revealed an increased GLUT-1-dependent glycolysis rate and spare respiratory capacity, suggesting that KFs behave according to a Warburg-like effect to meet the energy demand of increased cell viability. Moreover, enhanced GLUT-1 activity was shown in keloid tissues and KFs, and this regulates not only glycolytic rate but also the increased ROS levels in keloids. The GLUT-1 inhibitor suppressed cell viability, which is associated with the inhibited glycolytic function and reduced spare respiratory capacity in KFs. These results indicated that increased GLUT-1 expression in keloids may regulate the metabolic reprogramming and increased fibroblast proliferation in keloids (Figure 8).

Keloids are characterized by the accumulation of extracellular matrix and excess fibroblast proliferation, both of which are energy-intensive processes [44,45]. Compared to oxidative phosphorylation, glycolysis meets the energy demand more quickly, particularly in proliferating cells [32]. The adaptation of the cell’s energy requirements mirrors the high anabolic rate and low catabolic cellular of active fibroblasts [46]. In lung fibrosis, glycolytic enzymes and glucose transporters are abnormally high in human and murine fibroblasts [47]. In accordance with previous studies, our results demonstrated that KFs behave toward the Warburg effect with a partial increment of OXPHOS (high spare respiratory capacity) (Figure 1B,D). Enhanced glucose influx and increased glycolytic enzymes may be pathophysiological mechanisms in keloids.

In our experiments, GLUT-1 was expressed in keloids and it mediated enhancement of glycolysis in keloids. Changes in the metabolic state and oxidative stress can regulate GLUT-1 expression [48]. GLUT-1, responsible for glucose intake, contributes to cell division, differentiation, and transformation in response to stresses and various disease processes [49,50]. For example, many cancer cells overexpress GLUT-1 to fulfil the need for extra glucose intake because GLUT-1 can be activated quickly [51]. By inhibiting GLUT-1, the proliferation of cancer cells was decreased dramatically in the breast and non-small cell lung cancer [52]. In idiopathic pulmonary fibrosis, GLUT-1-dependent glycolysis is critical for parenchymal fibrosis and airway inflammation in a bleomycin-induced lung injury model [53]. Glycolysis was significantly suppressed after transfection with GLUT-1 shRNA or treatment with GLUT-1 inhibitor. The expression of collagen I and fibronectin in lung fibroblasts decreased accordingly [54]. In our study, KFs showed higher basal glycolytic rates, capacity, and glycolysis reserve, which provided higher glycolytic ATP output (Figure 1B). After exposure to WZB117 treatment, ECAR (including glycolysis, glycolytic capacity, glycolytic reserve) was selectively decreased in KFs but not in NFs (Figure 4). In contrast, after WZB117 treatment, OCR (including basal respiration, maximal respiration, and spare respiratory capacity) was decreased in NFs, not in KFs (Figure 5). Additionally, KFs showed augmented spare respiratory capacity compared to NFs (Figure 5F). These results indicated that KFs rely heavily on GLUT-1-mediated glycolysis whereas NFs are dependent on OXPHOS in fibroblast proliferation.

The KFs in our study showed a GLUT-1-mediated induction of ROS. Induction of ROS is implicated in the dysregulation of apoptosis and can damage both DNA and protein in keloid tissues [55]. Increased ROS production in KFs suggests an impaired cellular antioxidant system in keloids [41,42]. The presence of ROS, the byproduct of OXPHOS, indicates the aerobic metabolism works in the process [56]. A vicious circle of ROS-stimulated glucose uptake and glucose-stimulated ROS production can be triggered [57]. The increased ROS observed in KFs in this study is consistent with several previous reports [41,42], although the other study showed ROS generation was lower in KFs than in NFs. This discrepancy may result from the complex redox homeostasis mechanisms [36,58]. ROS promote fibroblast proliferation, myofibroblasts differentiation, EMT, and collagen deposition [59,60,61]. The glycolysis might be an adaptive shift to avoid the oxidative stress caused by OXPHOS [36]. Through NADPH generating from PPP and mediating transport of dehydroascorbic acid, glucose may serve as an antioxidant [62]. Evidence from previous works indicates that increased ROS levels stimulate increases in cellular glucose uptake at both slow and fast time scales. In L6 myoblasts, decreased GLUT-1 activity increases ROS levels, which suggests an ROS scavenging role for glucose [63]. In our study, basal ROS levels were increased in KFs. After WZB117 treatment, the ROS levels decreased in accordance with decreased fibroblasts proliferation (Figure 6 and Figure 7). Taken together, these results suggest that the inhibition of glucose influx may regulate the development of oxidative stress and coordinate the glucose metabolism in keloids.

Although the activation of glycolysis is observed in wound healing processes, the expression profiles of glycolytic enzymes are not consistent in different studies and fibrotic models. How GLUT-1 regulates glycolysis or OXPHOS in fibroblasts is seldom discussed in the literature. Vinaik et al. reported that keloids derived from patients with extensive burns showed upregulations of several glycolytic enzymes, including GLUT-1, HK2, PFK1, and PFK2 [64]. That study utilized the tissue and fibroblasts derived from burn-induced keloids, but not those from common keloids, which we were investigating. Moreover, in that study, the blocking molecules for GLU-1 were different in that study and in our study (shikonin and WZB117, respectively). Using the radiation-induced fibrosis in human and mouse models, Zhao et al. reported that the upregulation of glycolysis contributes to increased extracellular matrix deposition, as evidenced by that GLUT-1 inhibition with WZB117-reduced ECM accumulation [65]. However, that study included patients who received full-dose radiotherapy to the neck for cancer treatment, for which the fibrotic skin model was different from this study in patients with common keloids. The intrinsic factors of cancer itself and the exogenous radiation therapy in these patients confounded the experimental findings in that study.

For glycolysis in keloids, Wang et al. showed that in hypoxia-promoted proliferation, GLUT-1 expression was enhanced, as well as migration and collagen synthesis, and autophagy in KF [66]. However, in that study, no blocking measures for GLUT-1 or other glycolytic enzymes were performed. Our study showed, with GLUT-1 blockage by WZB117, that GLUT-1 mediated enhanced glycolysis and decreased ROS formation in KFs (Figure 4 and Figure 6, respectively) while increasing mitochondrial OXPHOs in NFs. Chen et al. investigated the inhibition of glycolysis-regulated KF proliferation through 2-DG, which is different from the inhibitor used in our study (WZB117). Moreover, that study measured the expression of several glycolytic enzymes, including HK2, PKM2, and LDHA, but not GLUT-1 [67]. Taken together, our study is distinct to others with ample experimental evidence showing that GLUT-1 mediated the enhanced glycolysis in keloids.

There are some limitations in this study. First, this study lacks a suitable keloid animal model that may limit the extrapolation of in vitro results to in vivo. Second, there were limited numbers of samples of tissues and tissue-derived fibroblasts (6 from keloids and 6 from healthy skin, 6 pairs). Relatively few keloid patients received surgical intervention due to a high likelihood of recurrence after surgery. Therefore, for research purposes, it is difficult to collect a large number of keloid samples, which is a general limitation of keloid research [2,4,68,69]. For this reason, not all the confounders could be matched, including age, gender, and the body sites, which may affect the intrinsic property of the fibroblasts in the present study. Further large-scale and animal studies may be required.

## 5. Conclusions

Our findings showed that human KFs exhibit Warburg-like effects of glycolysis in bioenergy utilization. By mediating glycolysis, GLUT-1 promoted KFs proliferation in concordance with increases in ROS and spare respiratory capacity. Whereas GLUT-1 has been identified as a therapeutic target for cancer [70], our study provides evidence that GLUT-1 is also a potential therapeutic target for keloids and other fibrotic diseases.

## Figures and Tables

**Table 1:** Demographic data of keloid and control subjects.

Subject	Age (Years)	Sex	Body Site	Duration	Ethnicity
Keloid 1	50	Male	Chest	2 years	Han
Keloid 2	58	Male	Chest	3 years	Han
Keloid 3	44	Female	Chest	2 years	Han
Keloid 4	65	Male	Chest	4 years	Han
Keloid 5	22	Female	Ear	3 years	Han
Keloid 6	26	Female	Shoulder	10 years	Han
Control 1	37	Male	Elbow	NA ^1^	Han
Control 2	51	Female	Inguinal area	NA	Han
Control 3	24	Female	Buttock	NA	Han
Control 4	41	Female	Forearm	NA	Han
Control 5	45	Male	Cheek	NA	Han
Control 6	45	Male	Chest	NA	Han

^1^ NA, not applicable.

**Table 2:** RT-PCR primer sequence.

Gene	Sequences (5′-3′)
GLUT-1	F: TGG CTC CGG TAT CGT CAA C R: GCT CGC TCC ACC ACA AAC A
Hexokinase	F: TGA AAA TCC GTA GTG GGA AAA AG R: TCA ATA GGA ATG GCG TAG ATC TTG
Phosphoglucose isomerase (GPI)	F: CCC AGG AGA CCA TCA CGA AT R: GCC GCC TGG AGA AAC CA
Phophofructokinase (PFK)	F: GCT GTA TTC AGA AGA GGG CAA AG R: GCA TGT GAC CCA GCA CGT T
Aldolase	F: CTC TAC CAG AAG GCG GAT GAT G R: AAC ACC GCC CTT GGA TTT G
Glyceraldehyde 3-phosphate dehydrogenase (GAPDH)	F: TGC ACC ACC AAC TGC TTA GC R: GGC ATG GAC TGT GGT CAT
Pyruvate kinase M2 (PKM2)	F: CCA TAA TCG TCC TCA CCA AGT CT R: GCA CGT GGG CGG TAT CTG
Pyruvate dehydrogenase kinase 1 (PDK1)	F: GCC TCT GGC TGG TTT TGG T R: CCT TGG AAG TAT TGT GCG TAA AGA
Lactate dehydrogenase (LDH)	F: GAG AGC ATG GCG ACT CAA GTG R: GGG ACG CCA GCA ATG TTC
β-actin	F: GAT GAG ATT GGC ATG GCT TT R: GTC ACC TTC ACC GTT CCA GT

## Data Availability

All data generated or analyzed during this study are included in this published article.
